# The "Transport Specificity Ratio": a structure-function tool to search the protein fold for loci that control transition state stability in membrane transport catalysis

**DOI:** 10.1186/1471-2091-5-16

**Published:** 2004-11-17

**Authors:** Steven C King

**Affiliations:** 1 Integrative Biosciences, Oregon Health & Science University, Portland, Oregon, 97239-3097, USA

## Abstract

**Background:**

In establishing structure-function relationships for membrane transport proteins, the interpretation of phenotypic changes can be problematic, owing to uncertainties in protein expression levels, sub-cellular localization, and protein-folding fidelity. A dual-label competitive transport assay called "Transport Specificity Ratio" (TSR) analysis has been developed that is simple to perform, and circumvents the "expression problem," providing a reliable TSR phenotype (a constant) for comparison to other transporters.

**Results:**

Using the *Escherichia coli *GABA (4-aminobutyrate) permease (GabP) as a model carrier, it is demonstrated that the TSR phenotype is largely independent of assay conditions, exhibiting: (i) indifference to the particular substrate concentrations used, (ii) indifference to extreme changes (40-fold) in transporter expression level, and within broad limits (iii) indifference to assay duration. The theoretical underpinnings of TSR analysis predict all of the above observations, supporting that TSR has (i) applicability in the analysis of membrane transport, and (ii) particular utility in the face of incomplete information on protein expression levels and initial reaction rate intervals (e.g., in high-throughput screening situations). The TSR was used to identify *gab *permease (GabP) variants that exhibit relative changes in catalytic specificity (k_cat_/K_m_) for [^14^C]GABA (4-aminobutyrate) versus [^3^H]NA (nipecotic acid).

**Conclusions:**

The TSR phenotype is an easily measured *constant *that reflects innate molecular properties of the transition state, and provides a reliable index of the difference in catalytic specificity that a carrier exhibits toward a particular pair of substrates. A change in the TSR phenotype, called a Δ(TSR), represents a specificity shift attributable to underlying changes in the intrinsic substrate binding energy (ΔG_b_) that translocation catalysts rely upon to decrease activation energy (). TSR analysis is therefore a structure-function tool that enables parsimonious scanning for positions in the protein fold that couple to the transition state, creating stability and thereby serving as functional determinants of catalytic power (efficiency, or specificity).

## Background

Structure-function analysis seeks to elucidate how the structural attributes of a protein serve its function. The function of a carrier protein is to *catalyse *transmembrane solute translocation. However, without a productive conspiracy among *catalysis-promoting residues *in the protein fold, transport proteins would be non-catalytic (i.e., unable to enhance transition state stability). Inasmuch as "... catalytic power will always appear as a result of increased transition state stabilization (lower free energy) ..." [1], a powerful addition to the structure-functionist's arsenal would be a generally applicable method that rapidly identifies sites in the protein fold that control transition state stability (i.e., that control the affinity of substrates for the transition state). What functional characteristics or properties might such a technique probe?

The structure-function technique would be required to provide a keyhole through which to view positions in the protein at which structural perturbations affect transition state binding energy, for it is well-appreciated that a catalyst creates transition state stability by binding substrates tightly in the transition state complex [2]. In fact, binding energy is thought to be significant to the exclusion of all else in carriers that catalyse translocation without any change in the covalent structure of the substrate [3]. Absent changes in substrate structure, it is implicit that the conformational motions of "alternating access" must produce a transition state complex in which the substrate is more tightly bound than in the initial Michaelis complex. Fundamentally, catalysis could not occur without this realization of additional binding energy in the transition state [4].

The present contribution demonstrates use of the Transport Specificity Ratio (TSR) as an analytical keyhole to capture an initial glimpse of positions in the protein fold where structural characteristics control the availability of transition state binding energy. Using the *Escherichia coli *GabP (*gab *permease) as a model carrier protein, salient properties and utilitarian features of TSR analysis are demonstrated. The TSR parameter is shown (i) to be calculated from an easily performed dual-label uptake experiment, and (ii) to depend *exclusively *upon changes in intrinsic substrate binding energy (ΔG_b_) realized in the transition state. Together these TSR properties should enable transport structure-functionists to obtain rapid, yet incisive, first-pass view of *positions in the protein fold where structure influences transition state stability and catalysis per se.*

## Results

### Effect of substrate concentration on the TSR

**Figure 1:**
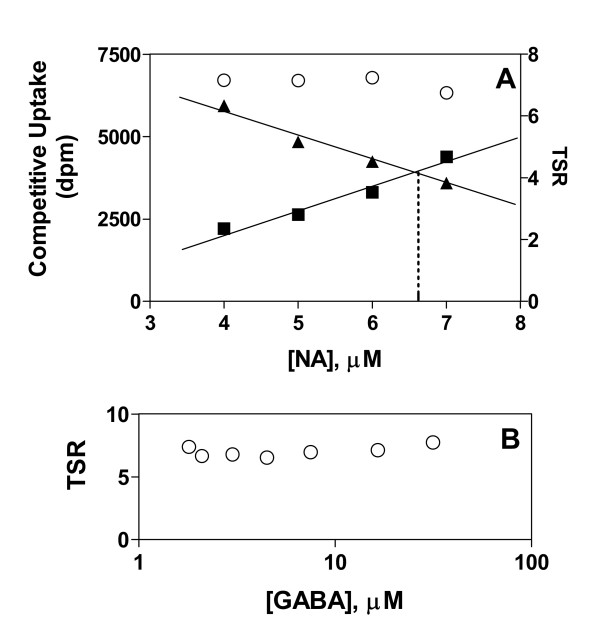
**Results from TSR analysis are valid across a broad range of competing-substrate concentration ratios **The Transport Specificity Ratio (TSR) is calculated using results from a dual-label competitive uptake assay in which structurally distinct, labelled substrates are allowed to compete for transport at the same active site. ***Panel A: ***Mixtures of 10 μM [^3^H]NA (0.6 μCi/ml) and 10 μM [^14^C]GABA (0.2 μCi/mL) were prepared such that [NA] + [GABA] = 10 μM. *E. coli *strains SK105 (GabP-positive) and SK45 (GabP-negative) were exposed in parallel experiments for 10 seconds at 30°C to substrate mixtures containing the indicated concentrations of [^3^H]NA. The GabP-dependent (SK105 minus SK45) uptake of either [^3^H]NA (■) or [^14^C]GABA (▲) may be read from the left-side ordinate. The calculated TSR (Equation. 6) may be read from the right-side ordinate (○). ***Panel B: ***The substrate concentrations were varied in constant proportion such that the GABA concentration (ranging from 1.8–31.5 μM) was always 42.9 percent of the NA concentration (ranging from 4.2–73.4 μM). The radiochemical concentrations for [^3^H]NA and [^14^C]GABA were 0.23 μCi/ml and 0.03 μCi/ml, respectively. The indicated concentration ranges produce about 50 percent combined active site occupancy (bound GABA plus NA) – since the affinities for GABA and NA are 40 μM and 200 μM, respectively [25].

TSR analysis as implemented in present examples consists of a dual-substrate transport assay in which [^14^C]GABA and [^3^H]NA compete for uptake at the GabP active site. Therefore as a practical matter it is necessary to establish conditions under which an adequate signal may be obtained from both isotope channels. This can be accomplished empirically by mixing the labelled substrates in different proportions (Fig. 1A). In the range from 1–10 μM (below expected K_m _for either substrate) the trading of [^3^H]NA for [^14^C]GABA is expected to substantially alter the fraction of active sites occupied by GABA versus NA Nevertheless, it is clear that the calculated TSR parameter is indifferent to the precise substrate concentration ratio. Moreover, at a fixed substrate ratio (7 parts NA to 3 parts GABA), the absolute substrate concentrations may also be varied over a wide range (here 17.5-fold) without affecting the calculated TSR parameter (Fig. 1B).

Although these data (as well as the underlying theory) indicate that there is great latitude in choosing substrate concentrations for TSR measurements, it is nevertheless pragmatic to select robust initial velocity conditions wherein the substrate concentration ratio is such that equal disintegration rates are seen in both isotope channels (broken line) when the control (e.g., wild type) transporter is studied. Variant transporters, exhibiting relative increases or decreases in specificity for the two substrates, will then be easily visualized as an inequality between the disintegration rates seen in the two isotope channels (so that the data are no longer graphically superimposed).

### Effect of protein expression level on the TSR

**Figure 2:**
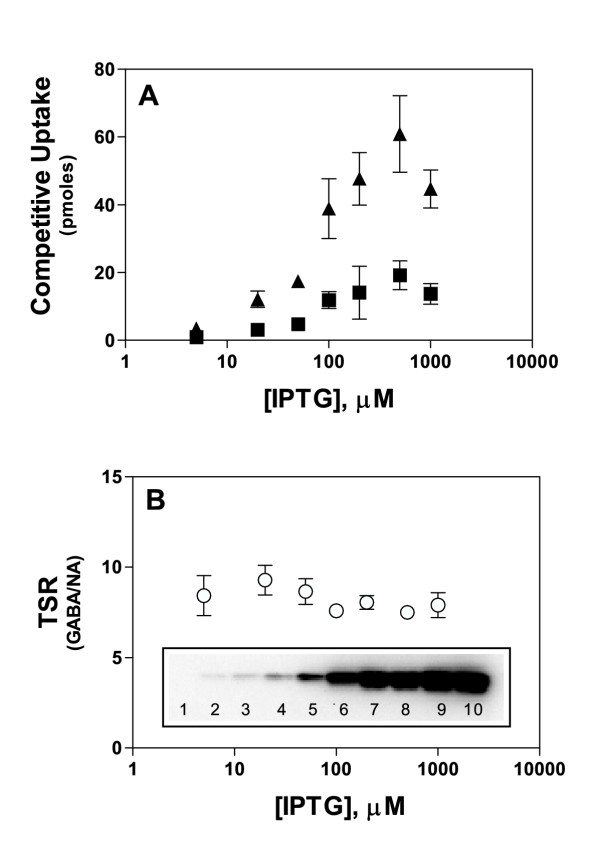
**Results from TSR analysis are valid across a broad range of carrier expression levels ***E. coli *strains SK11 (GabP-positive) and SK45 (GabP-negative) were grown to early logarithmic phase as described in *Methods *except that expression was induced by exposing cultures to the indicated IPTG concentrations. The cells were washed with 100 mM potassium phosphate buffer (pH 7.0), and dual-label competitive transport reactions were initiated by exposing the cells to 7 μM [^3^H]NA (0.42 μCi/ml) and 3 μM [^14^C]GABA (0.06 μCi/ml) for 10 seconds (initial rate) at 30°C. Error bars represent the S.E.M. (n = 3). **Panel A. **GabP-dependent uptake (SK11 signal minus SK45 signal) of either [^3^H]NA (■) or [^14^C]GABA (▲). **Panel B. **Transport Specificity Ratio (GABA/NA). **Inset. **Immunoblot of plasma membrane vesicle protein (2 μg per lane) probed with an anti-pentaHis mAb and developed with a chemiluminiscent alkaline phosphatase substrate (see *Methods*). **Lane 1: **Membranes from *E. coli *strain SK45 (GabP-negative). **Lanes 2–10: **Membranes from *E. coli *SK11 (GabP-positive) grown in the presence of 2, 5, 10, 20, 50, 100, 200, 500, or 1000 μM IPTG, respectively.

When distinct transporter variants are studied, it frequently is the case that the strains will express the variants at distinct and unpredictable levels in the plasma membrane, complicating the interpretation of any observed differences in transport velocity. In order to discern how expression-level affects the TSR analysis, IPTG was used to induce to differing levels the *lac*-controlled expression of the plasmid-borne GabP gene. Growth in the presence of increasing IPTG concentrations caused the uptake of [^3^H]NA and [^14^C]GABA to increase in proportion to the GabP expression-level (Fig. 2A), which was monitored by immunoblot (Fig. 2B, inset). Although single-substrate uptake and expression varied over a 40-fold range, the calculated TSR parameter held steady (Fig. 2B), indicating that differing expression levels would have a minimal effect on results obtained by TSR analysis.

### Effect of assay end-point on the dual-substrate mole ratio

**Figure 3:**
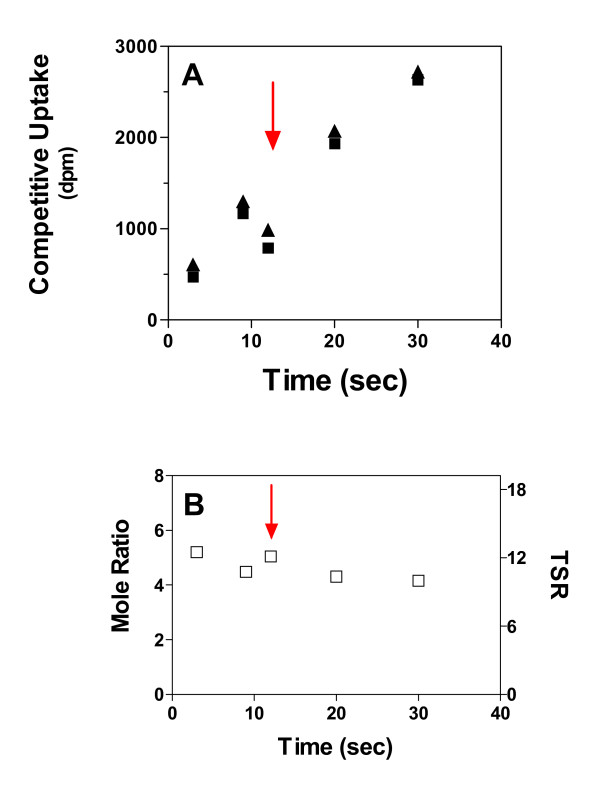
**Results from TSR analysis are valid across a broad range of reaction times ***E. coli *strain SK11 (GabP-positive) was exposed simultaneously to 6 μM [^3^H]NA (0.42 Ci/ml) and 4 μM [^14^C]GABA (0.06 Ci/ml) for the indicated times at 30°C. Parallel experiments were carried out in the presence of 2 mM GABA, which was included to block the GabP. **Panel A **shows the GabP-dependent component of competitive uptake (difference between the parallel experiments) over a 10-fold time range. The **red arrow **indicates a probable mechanical error, causing low uptake inconsistent with other points on the curve. The **Panel B **shows the GABA to NA mole ratio (left-side ordinate) calculated from data shown in the Panel A. The associated TSR values may be read from the right-side ordinate. The red arrow has the same meaning as in the Panel A, and serves here to emphasize the reliability of the TSR analysis, which has self-correcting properties that compensate for many routine sample processing problems that may cause inconsistency in times or volumes (see discussion).

Single-substrate transport velocities may be estimated from the slope of the initial-rate segment of an uptake time course (Fig. 3). Unlike single substrate uptakes, which are linearly affected by deviations from the intended stopping time, the dual-substrate "mole ratio" is time-independent across the linear range studied. Thus, mechanical errors affecting the "stop-time" should be largely self-correcting. Indeed, the red arrow (Fig. 3A) marks the position of an indeterminate error, wherein the single-substrate data points are off the curve suggested by the remaining data. This error is seen to "self-correct" in the dual-label "mole ratio" and TSR calculations (Fig. 3B, red arrow), indicating that dual-label ratio parameters can be more reliably estimated than can single-substrate velocities. In fact, many errors in time and volume will "self-correct" in the TSR calculation (see Discussion section).

### Assignment of TSR phenotypes to GabP variants

**Figure 4:**
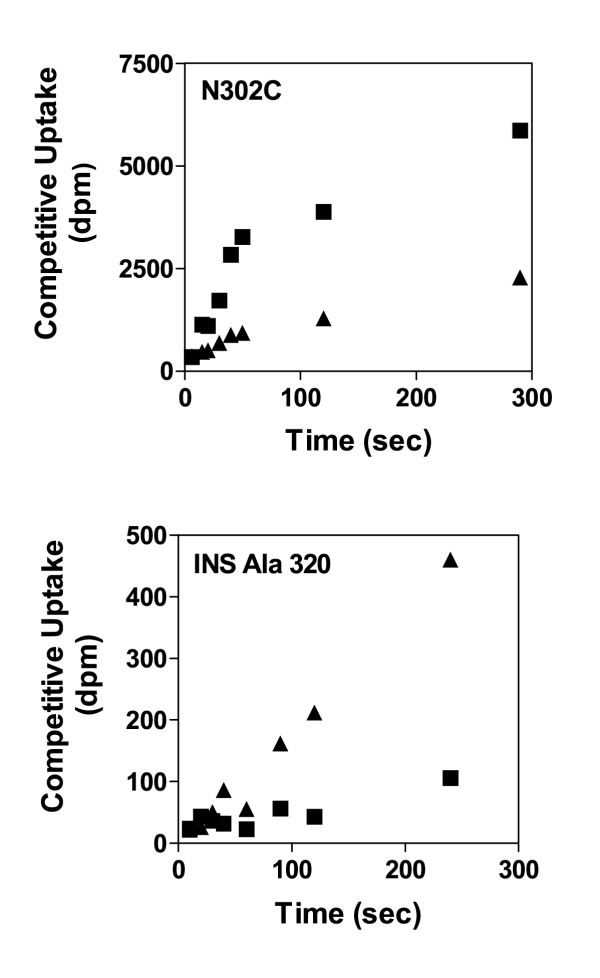
**Variants of the *E. coli *GabP that exhibit Δ(TSR) phenotypes **Using data analogous to Figure 1, the concentrations of competing substrates were adjusted empirically such that the initial rates of label accumulation were superimposed for *E. coli *strains expressing the "control" *gab *permease (GabP). As a result, any separation between initial rate uptake curves for [^14^C]GABA (▲) and [^3^H]NA (■) provides a highly intuitive visual representation of a Δ(TSR) phenotype. **Panel N302C **shows TSR analysis of the single-Cys GabP variant, N302C. Compared to the Cys-less GabP control (TSR = 8) for which the initial label accumulation rates are superimposed [5], the N302C shows a relative increase in the specificity for NA with a calculated TSR of 2.5. The **Panel INS Ala 320 **shows TSR analysis of the GabP variant, INS Ala 320, which has an extra alanine residue inserted at position 320. Compared to the wild type GabP control (TSR = 4) for which the initial label accumulation rates are superimposed [6], the INS Ala 320 exhibits a relative increase in specificity for GABA (i.e., opposite of the Panel N302C) with a calculated TSR of 16.

When assay conditions conform to recommendations (Fig. 1), then transporters serving as the "parental control" will exhibit superimposed initial rate segments on the uptake time courses for accumulation of the [^3^H] and [^14^C] labels [5,6]. Clearly, the GabP variants shown in Figure 4 do not exhibit superimposed initial rate segments, indicating in a highly intuitive visual fashion that the TSR phenotype for these variants will differ from their respective parent transporters. Compared to its Cys-less parent (control TSR = 8), the single-Cys variant, N302C, exhibits a relative increase in preference for NA (TSR = 2.5). Compared to its wild type parent (control TSR = 4), the INS Ala 320 variant (with an extra alanine residue inserted at position 320) exhibits a relative increase in preference for GABA (TSR = 16).

## Discussion

### TSR phenotyping derives from a concrete definition of catalysis

In order to initiate development of a structure-function relationship for translocation *catalysis *by GabP [5,6], it was useful to adopt a formalism that describes *catalysis *in concrete terms [7] so that structural perturbations affecting *catalysis *might likewise be described in terms of a concrete (quantifiable) phenotype – the TSR. Fundamental to TSR analysis is the notion that transport catalysts use substrate binding energy to lower the translocation energy barrier (activation energy) [2]. Equation 1 states that the catalysed activation energy () is lower than the non-catalyzed activation energy () by an amount equal to the intrinsic substrate binding energy, ΔG_b _(algebraically negative).



**Figure 5:**
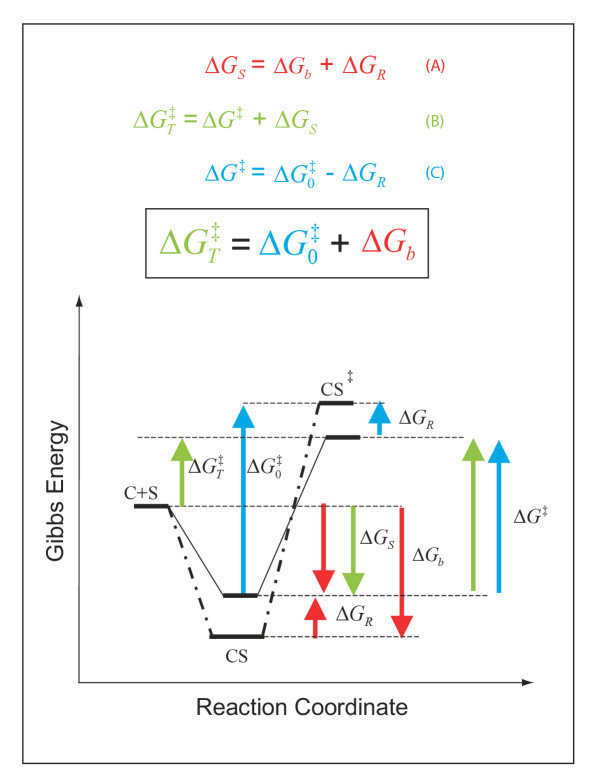
**Changes in catalytic specificity (k_cat_/K_m_) reflect underlying changes in transition state binding energy (ΔG_b_) **In this description of catalysis, (i) the magnitude of the non-catalysed activation energy () does not depend on a favourable protein-substrate interaction in the transition state, (ii) the catalysed *translocation energy barrier *is taken as the Gibbs Energy difference () between the free reactants (C + S) and the transition state complex (*CS*^‡^), and (iii) intrinsic substrate binding energy is recognizable as the decisive factor in transition state stabilization. Thus, translocation catalysts (C) will use intrinsic substrate binding energy (Δ*G*_*b*_) to stabilize the transition state (CS^‡^). The role of Δ*G*_*b *_in lowering the transition state energy barrier compared to a non-catalyzed reaction () may be deduced with aid from the accompanying energy diagrams, which emphasize several instances wherein the thermodynamic distance represented by one coloured arrow equals the summed distance represented by two shorter arrows of the same colour. The illustrated thermodynamic relationships are restated (with proper attention to sign convention) in equations A (red), B (green), and C (blue). Substituting A and C into B yields the fundamental relationship,  (boxed), which says that the uncatalysed activation energy (, algebraically positive) is diminished by intrinsic substrate binding energy, Δ*G*_*b *_(algebraically negative), which is the underlying parameter that TSR analysis probes (Eqn. 9). **Note: **These energy diagrams compare non-catalytic (dots and dashes) and catalytic (solid line) proteins. Imposition of a binding-averse interaction (Δ*G*_*R*_) is seen to *de-stabilize *the Michaelis complex (CS, red arrows) in the catalytic protein. Subsequent attainment of favourable transition state complementarity (i.e., via conformational transitions that relieve Δ*G*_*R *_, blue arrows) results in use of binding energy to *stabilize *the transition state complex (CS^‡^). This internal ''give-and-take,'' involving Δ*G*_*R *_is reflected in its algebraic cancellation when equations A, B, and C are combined to yield the boxed equation (text Eqn. 1), which says that intrinsic substrate binding energy decreases the energy barrier () for a translocation reaction carried out from solution (i.e., directly from the *free *carrier and substrate (C + S) to the transition state). When C and S are free in solution, the effective second-order rate constant associated with  is k_cat_/K_m_, the specificity parameter compared in the dual-substrate TSR analysis (Equation.5). That k_cat_/K_m _should be associated with the free reactants may be appreciated by considering the Michaelis-Menten Equation when S << K_m_, and CS complexes do not exist in appreciable amounts (see *Discussion*).

Importantly, Equation 1 (justified by Fig. 5) tells us that to screen for changes in catalytic power (barrier height) per se, *one must find an easily measured signal that reports on changes in the intrinsic substrate binding energy (ΔG_b_) used to stabilize the transition state. *That the Transport Specificity Ratio (TSR) analysis fulfills this requirement may be shown as follows.

### The Michaelis-Menten equation in two variables

The velocity (*v*) of a simple translocation reaction, carried out from solution (C + S → Products), is governed by a second-order rate law (Equation 2), wherein the apparent second-order rate constant is *k*.

*v* = *k*[*C*][*S*]     (2)

Free carrier and substrate (C + S) are dominant under non-saturating, second-order conditions (i.e., [S] << K_m_), wherein the familiar Michaelis-Menten relationship (Equation 3) reduces to the form of a second-order rate law (Equation 4), and the apparent rate constant may be evaluated as k = k_cat_/K_m _(units M^-1^sec^-1^).





Although Equation 4 may appear to be a special case, it is actually a generally valid alternative form of the Michaelis-Menten Equation that is little used because it contains two variables, [C] and [S]. Equation 4 is valid at all substrate concentrations, producing the same saturating substrate-velocity curve as Equation 3 (since [C] goes to zero as [S] goes to infinity). The alternative Michaelis-Menten form turns out to be very useful for analysing the uptake of two labelled substrates that compete for transport at the same active site.

### Competing substrates equilibrate with the same free carrier concentration

Consider the *E. coli *GabP exposed simultaneously to arbitrary concentrations of its transported substrates [8,9], [^14^C]GABA and [^3^H]NA. These competing substrates, present simultaneously in the same reaction vessel, will necessarily be in equilibrium with precisely the same concentration of *free *carrier (but unknown concentrations of carrier-substrate complexes), allowing algebraic elimination of [C] (Equation 5) when a ratio is taken between two instances of Equation 4 (one for each substrate).



### Catalytic specificity reflects the translocation energy barrier height

That Equation 5 contains the ratio of a pair of (k_cat_/K_m_) values has two consequences. First, since (k_cat_/K_m_) is formally a measure of catalytic *specificity *[7,10], we may recast Equation 5 succinctly in terms of the Transport *Specificity *Ratio (TSR) parameter.



Secondly, since (k_cat_/K_m_) is an apparent rate constant (see above), transition state theory holds that its value depends upon the height of the translocation energy barrier () as indicated by this logarithmic form of Eyring's Equation (Equation 7),



wherein *k *is the Boltzman constant, *h *is the Planck constant, R is the gas constant, T is the absolute temperature, and a transmission coefficient of unity is assumed.

### Specificity ratios depend only upon intrinsic binding energy differences

If catalytic specificity (k_cat_/K_m_) depends upon , then by implication the TSR must be related to the intrinsic substrate binding energy – as becomes evident when Equations 1 and 7 are combined,



Equation 8 shows that k_cat_/K_m _(synonymous with catalytic power, specificity, and efficiency) varies with the amount of transition state stabilization afforded by ΔG_b_, which is the intrinsic substrate binding energy (algebraically negative). Taking the ratio between two instances of Equation 8 (e.g., for the two competing substrates, GABA and NA), and combining terms, we obtain



Equation 9 indicates that an experimentally observed change in the TSR parameter would require a change in the underlying intrinsic substrate binding energies that determine the relative height of the translocation energy barriers for two substrates competing at the same active site.

### The TSR reflects a change in substrate affinity for the transition state

**Figure 6:**
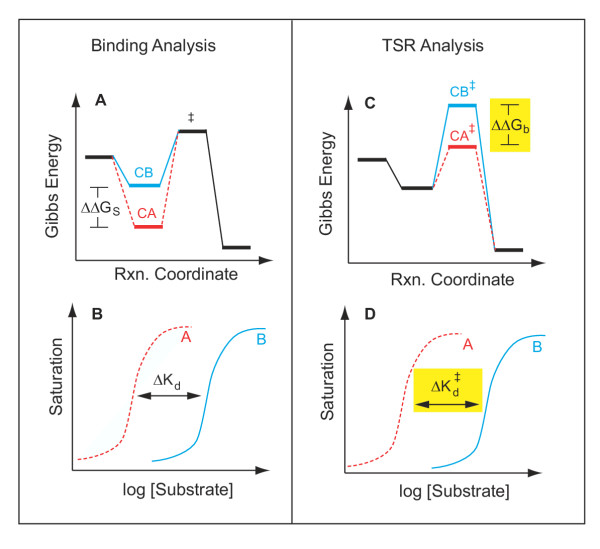
**Comparison of equilibrium binding versus TSR analysis **Envisage a catalytic protein interacting with two substrates (or substrate analogs), one exhibiting high-affinity binding (dashed RED line), and the other low-affinity binding (solid BLUE line). Equilibrium binding to the stable Michaelis complex (LEFT, Panels A and B) would produce concentration-dependent saturation of the binding site (Panel B). From the observed affinity difference (ΔK_d_) between the two substrates, one can calculate a corresponding difference in binding energy, ΔΔG_S _(Panel A), for the two substrates interacting with the stable Michaelis complex at the bottom of the reaction coordinate. In contrast, information on the interaction of substrates at the reaction coordinate peak would require a study of binding to the unstable transition state (RIGHT, Panels C and D). Unfortunately, due to the high energy-level and transient nature and of the transition state (denoted by ‡), the relevant binding experiment (Panel D) is technically impossible. However, TSR analysis allows direct calculation (Equation 9) of the transition state binding energy difference, ΔΔG_b _(Panel C, yellow) between two competing substrates, A and B. A change in the TSR phenotype, or Δ(TSR), thus provides evidence for a change in the graphical separation distance,  (Panel D, yellow), for the "impossible experiment" on substrate binding to the unstable transition state. Thus, observation of a Δ(TSR) phenotype reflects underlying structural changes that affect binding discrimination between substrates A and B in the transition state, which are of interest because transition state binding interactions create transport catalysis [2–4, 7] by lowering the activation energy, , and increasing k_cat_/K_m_. In summary, the equilibrium binding experiment depicted on the left does not address catalysis *per se*, whereas the TSR experiment depicted on the right does.

Figure 6 emphasizes that in the comparison of two substrates, the TSR reflects a difference in substrate affinities for the transition state (at the reaction coordinate peak). This contrasts with true equilibrium binding measurements, which reflect substrate affinities in the initial Michaelis complex (at the reaction coordinate bottom). These affinities are characterized by the dissociation constants, K_d _and , which describe the equilibrium position of reactions leading to formation of free reactants from either the Michaelis complex (CS ↔ C + S) or the transition state complex (CS^‡ ^↔ C + S), respectively. Inasmuch as equilibrium constants (e.g., ) are always determined by Gibbs energy differences (e.g., Δ*G*_*b*_ = -*RT* ln ), it follows (Fig. 6, yellow shading) that a change in transition state binding energy (ΔΔG_b_) reflects a change in the midpoint separation () between hypothetical curves that describe binding of two test substrates (A and B) to the transition state. Structural features that affect the "tightness" of transition state binding will alter the translocation energy barrier height (Equation 1), which determines synonymously the *catalytic power, efficiency, or specificity of a transporter.*

### The TSR phenotype is a constant

Unlike first-pass analytical methods that rely on the signal from one labelled substrate, the herein described dual-label analysis leads directly to the TSR parameter – *a constant *(Equation 9). Constants are intrinsically stable and reliable, reflecting fundamental reaction characteristics that survive changes in ambient conditions (provided temperature and pressure can be held constant). The unique stability and fundamental nature of the TSR phenotype will make it particularly valuable for first-pass analysis in high-throughput screening situations, wherein protein expression levels, duration of the initial rate time course, and degree of saturation by the chosen substrate concentration may be inconsistent across large numbers of transporter variants with differing functional characteristics. This reliability is demonstrated using the *E. coli *GABA permease (GabP) as a model translocation catalyst. Overall the present study makes clear that the dual-label TSR analysis is insulated remarkably well from many uncontrolled variables that can often compromise the validity of assays that use a single label.

### TSR analysis is valid at arbitrary site-saturation levels

Figure 1 shows that the TSR did not change when GabP was exposed to [^14^C]GABA and [^3^H]NA in different proportions, or in fixed proportion over a broad concentration range (Fig. 1B). Indeed, the form of Equation 6 suggests that the velocity ratio should self-adjust continuously with changes in the dual-substrate concentration ratio (since the TSR and its component parts, k_cat _and K_m_, are all constants). Thus, arbitrary carrier saturation levels are not expected to compromise TSR measurements. Since uncharacterized mutant collections may be expected to contain transporter variants with highly divergent K_m _values, the saturation-independence of TSR analysis should be of value in high-throughput screening situations where little kinetic information may be available to guide the choice of assay conditions. However, to be of general value the results obtained with GabP must extrapolate to other transporters.

Why the deceptively simple TSR analysis should have broad applicability can be understood from further consideration of Figure 1. When substrate concentrations are varied, carrier saturation levels change, producing new complexes (e.g., [C· GABA] and [C· NA]) in changing proportions. While manipulating these complexes affects single-substrate uptake velocities significantly (Fig. 1), the TSR calculated from these velocities is unaffected because these particular complexes (and complexes of any arbitrary number and description) never have a role in determining the equilibrium – energetic distance () – between the free reactants (C + S) and the transition state (CS^‡^). This fundamental reality can also be appreciated from the perspective that under non-saturating conditions ([S] << K_m_), there are no complexes to consider ([C] = C_total_), and thus even complicated mechanisms reduce to the simple case (Equation 4) in which the reaction proceeds directly from the free reactants in solution to the transition state (C + S → Products). Thus, the simple second-order reaction scheme, C + S → Products, will probably never be "too simple" for the purpose of performing the TSR analysis – even though complicated transport kinetics will feature many complexes that TSR analysis seems to ignore. In truth, the missing complexes are merely irrelevant (not ignored) to the value of  (Fig. 5) since these complexes would always lie energetically between (or below) the free reactants (C + S) and the transition state complex (CS^‡^).

### TSR reliability stems from self-correcting properties

It is worth mentioning that TSR analysis has "fool-proof" qualities that derive from its inherent insensitivity to several sources of error that can seriously compromise transport measurements that rely upon a single labelled substrate. TSR calculations may be expected to "self-correct" any sources of error that have proportionally the same effect on the measurement of both isotopes – for such errors cannot affect the isotope ratio used to calculate the TSR parameter.

Figure 3, for example, shows that whereas stop-times affect single-isotope uptake signals in linear fashion, the dual-substrate mole ratio (and TSR calculation) is hardly affected meaning that TSR analysis is inherently insensitive to vast timing errors. In the experiment shown, stopping at arbitrary times across 10-fold range would have impacted the TSR calculation very little. Likewise, most sample handling errors (e.g., pipetting, filtering) will tend to affect both isotopes proportionally so that whereas the single isotope uptakes are affected linearly, the TSR calculation is preserved (Fig. 3, red arrow). Perhaps most importantly, TSR analysis can correct for sample-to-sample variations in protein expression-level (Fig. 2).

In order to demonstrate the expression-independent nature of the TSR parameter, IPTG was used to simulate the wide range of expression levels (40-fold) that might be encountered in an uncharacterized collection of transporter variants. Whereas the single-isotope signals (Fig. 2A) are seen to vary directly with GabP expression, the dual-isotope TSR phenotype (Fig. 2B) varies little. This expression-independent behaviour fully complies with theoretical expectations since (i) the carrier concentration was algebraically eliminated (Equation 5), and (ii) TSR is a "constant" (Equation 9), reflecting fundamental molecular properties of carrier-substrate interaction that do not depend upon the number of carrier molecules expressed in the membrane. The ability to rapidly evaluate a TSR phenotype, formally an expression-independent *constant*, should be of considerable practical significance for high-throughput screening operations wherein carrier expression levels could be both highly variable and impractical to document in real-time.

Since TSR phenotypes are expression-independent, structure-function information gleaned from a rapid first-pass screen will remain valid irrespective of results that might be obtained from a subsequent immunoblot analysis. Immunoblots do not in any event determine C_total _in the sense desired for meaningful kinetic characterization, which assumes (Equation 3) that C_total _consists entirely of active molecules. The possibility of partial denaturation precludes assigning a molecular interpretation to shifts in either *velocity *or V_max_. In contrast, TSR analysis is unaffected by the presence of inactive molecules, and theoretically will always report reliably on the innate *specificity *properties of the active site per se – even if the measured signal emanates from a minor fraction of the carrier molecules visualized on an immunoblot.

### TSR analysis detects "relative" specificity shifts

The TSR method is inherently capable of detecting new phenotypes that reflect relative specificity shifts favouring either test substrate (Fig. 4). Preliminary to these experiments, dual-substrate ratios were empirically adjusted so that control strains would exhibit superimposed [^14^C] and [^3^H] initial rate segments in their uptake time course (shown elsewhere, [5,6]). Plainly, the test cases in Figure 4 do not exhibit superimposed dual-isotope time courses, indicating two distinct Δ(TSR) phenotypes – one relatively favouring NA (Panel N302C), and the other relatively favouring GABA (Panel INS Ala 320).

The Δ(TSR) phenotypes illustrated in Fig. 4 are distinct from one another (and distinct from the control) because there are *relative differences *in transition state binding energies (Equation 9) that can be visually represented as a change in the *relative position *of (separation between) the hypothetical binding isotherms for either substrate (Fig. 6D). It is important to emphasize, however, that TSR analysis does not address the absolute magnitude of transition state binding energy shifts, nor the absolute magnitude of shifts in the binding curve midpoint ( shifts). This point is important, and can be illustrated by examining the implications of the figure 4 time courses in more detail.

Calculated TSR values for the N302C and INS Ala 320 variants are, respectively about 2.5 and 16. That these numbers are both greater than 1 indicates (Equation 9) that the hypothetical transition state binding isotherm for GABA would lie to the left (i.e., like the red curve in Fig. 6D) of the NA curve in both variants. If the measured TSR had been unity, then the hypothetical binding curves would be superimposed. If the measured TSR had been below 1, then the NA binding isotherm would lie to the left. Thus, the N302C time course with squares increasing faster than triangles (Fig. 4, Panel N302C) does not indicate an absolute preference favouring NA over GABA, but rather a squeezing down of separation between midpoints on the hypothetical transition state binding isotherms for GABA and NA (relative to the separation in the Cys-less control–TSR = 8). The INS Ala 320 time course with triangles increasing faster than squares (Fig. 4, Panel INS Ala 320) indicates an increase in the separation between midpoints on the hypothetical binding isotherms (relative to the separation in the wild type control – TSR = 4).

Although the ability to measure only *relative *changes in specificity has limitations, the reader will appreciate that to a mathematical certainty no relative shift can occur in the absence of one or more *absolute *shifts. TSR analysis thus provides an analytical keyhole through which to scan [5,6] the protein fold, looking for Δ(TSR) phenotypes indicative of *loci at which transition state stability can be controlled by amino acid side-chain structure. *This conclusion cuts directly to the essence of what a translocation catalyst does – fairly respectable performance for a first-pass, rapid-screening methodology, which minimally can consist of as little as a single datum point for each variant transporter screened.

It is to be noted that since absolute specificity changes can occur in the absence of a relative specificity shift (i.e., equal displacement of the binding isotherms for both substrates), some catalytic residues may be detectable only by more complicated kinetic studies, or possibly through independent TSR experiments with structurally distinct substrate pairs. Since the TSR parameter is a *constant *that characterizes how the transition state interacts with a particular pair of substrates, different results may be expected with structurally distinct substrate pairs. However, the observation of a Δ(TSR) phenotype always means the same thing – there has been a change in the transition state stability for translocation of one or both substrates.

### TSR analysis enables a broad search for the seat(s) of catalytic power

Apart from its delightful simplicity and self-correcting behaviour, the TSR (or rather the ability to observe Δ(TSR) phenotypes) is also attractive as a facile means of expanding interest in "coupled promoting motions" that are networked together in support of catalysis [11]. Such networks (i) are evolutionarily conserved, (ii) undergo conformational oscillations on the timescale of (in *synchrony *with) the catalyzed reaction, and (iii) collectively can make million-fold contributions to catalytic specificity (transition state stabilization) even though their locations are spatially distant from the active site in enzymes of known structure (e.g., dihydrofolate reductase [1,12-14]]; aspartate aminotransferase [15]). Inasmuch as Equation 8 says that *specificity* (k_cat_/K_m_) is a function of transition state stabilization ( + Δ*G*_*b*_), phenotypic changes in the TSR phenotype should report on structural perturbations that compromise as yet undiscovered networks that couple energetically to the transition state.

Inquiry along this line follows up on a prominent message emerging from recent literature on enzymatic catalysis: structural elements delocalized from the active site can enhance catalytic power (k_cat_/K_m_) by many orders of magnitude [1,11] – so that any understanding of enzymatic catalysis based on consideration of the active site in isolation may now be considered incomplete. This *delocalization *of catalytic power will in all likelihood be true of transport catalysis as well – but even more so since carriers lack a traditional active site (no covalent change in substrate structure). The catalytic power (specificity) of a carrier must therefore derive entirely from *conformational motions *that lead to tighter ligand binding in the transition state. This crucial catalytic increment in ligand binding energy could be *localized *within a binding pocket only to the extent that it is possible for a conformational transition to increase carrier-substrate complementarity without at the same time causing a change in conformational energy (structural stability). If conformational energy changes as the transition state forms, then one expects obligatory partitioning of transition state binding energy among multiple interactions (steric, electrostatic, hydrogen-bonding, or solvation forces) at highly *delocalized *positions throughout the protein fold. Since plasma membrane transport proteins consist mainly of bundled helices that exhibit rigid-body behaviour [16,17] it is unlikely that conformational remodelling of helix-helix interfaces could occur without changing conformational energy. Thus, localized control of translocation specificity (catalytic power) is also quite unlikely, and instead the determinants of specificity ought to be distributed rather broadly at *dynamic interfaces *throughout the helix-rich structure (a hypothesis that should be broadly testable by TSR scanning approaches [5,6]).

### "Alternating Access" vitiates feasibility of localized specificity control

Carrier proteins exhibit a compact tertiary structure in which tightly bundled helical segments span the membrane in a serpentine zig-zag fashion with extensive helix-helix contacts throughout [18,19]. The conformational transitions of "alternating access" (i.e., the general mechanism by which carriers expose a binding site alternately to one side of the membrane and then the other) thus proceed with extensive rigid-body remodelling of helix-helix interfaces. At some point in the translocation process the initial Michaelis complex (CS) is converted to a transition state complex (CS^‡^) with realization of additional binding energy (i.e., a change in the chemical potential of bound ligand), which creates catalysis. But what part(s) of the protein structure may be held to account for this pro-catalytic increment in binding energy?

Although not concerned with catalysis per se, Tanford set down clear principles from which we can infer that the binding energy used for transition state stabilization should have two *obligatory *sources in a helix-rich translocation catalyst – one source being dynamic motions in the protein fold. Tanford understood that with "...both translocation and change in chemical potential [of bound ligand] occurring in synchrony ..." [20] via helical tilts and twists, "it is not possible to separate free energy changes attributable to direct bonding to the proteins from free energy changes attributable to rearrangement of the protein structure that may accompany the binding process." [21]. Indeed, Benkovic's recent work on dihydrofolate reductase has provided the first visualization (molecular dynamics simulation) of the dynamic processes by which spatially distal motions in the protein fold can be coupled *synchronously *with active site rearrangements to create greater transition state stability.

Thus when distal, energy-changing, conformational motions occur in *synchrony *with (i.e., on the same timescale as) reconfiguration of the bound ligand (as with translocation or covalent structural change), then delocalized contributions to transition state stability *must *occur. Although the details may vary from case to case, the operable mechanisms will probably be conceptually similar to those that now have been visualized in dynamic simulations as "...coupled promoting motions extending throughout the protein and ligands, where promoting motions refer to equilibrium, thermally averaged conformational changes along the collective reaction coordinate leading to configurations conducive to the reaction." [1].

Importantly, the chance occurrence of a favourable dynamic coupling interaction would be accompanied by an evolutionarily selectable substrate specificity (k_cat_/K_m_) shift, suggesting that delocalized coupled promoting motions should be the rule rather than the exception. Engineered structural manipulations that interfere with the operation of coupled networks should impact k_cat_/K_m _such that elements of these networks may be rapidly detectable by TSR analysis. Such use of TSR analysis prompts re-examination of philosophical issues concerning the efficacy of mutagenesis in structure-function analysis.

### The ambiguity of mutagenesis reflects a truth about catalysis

Many, including this author, have cautioned that mutagenesis is associated with built-in thermodynamic constraints that produce confounding *ambiguity *when the *stated desire *is to use engineered structural perturbations as a means to identify residues of an active site [22,23]. However, it needs to be emphasized that Nature, also bound by thermodynamic constraints, relies continuously upon natural selection, taking meaningful advantage of the same *ambiguity *that the structure-functionist traditionally bemoans. This thermodynamic ambiguity provides that spontaneous mutations affecting structure at locations spatially distinct from the active site may nevertheless have pro-catalytic or anti-catalytic effects that become subject to natural selection. The evolutionary accumulation and coupling together of such pro-catalytic sites has produced now recognizable "networks of coupled promoting motions" that exist far from the active site, yet operate in synchrony with it to promote catalysis [1,11].

That catalysis [1,11] and energy transduction [20-22] appear to rely upon coupled motions in the protein fold raises a question as to whether the structure-function field might benefit from a change in its outlook on the *ambiguous *characteristics of mutagenesis, henceforth treating *ambiguity *as a friend that can reveal the location of coupled networks. Widely perceived as a shortcoming, this *ambiguity* turns out to be an accurate reflection of how Nature uses the protein fold to boost catalytic power. It simply is not the case that a kcal of transition state stabilization emanating from a few residues in the active site is worth (by some visceral rationale) more than a kcal of transition state stabilization emanating from the protein fold.

## Conclusions

TSR analysis is a remarkably simple dual-substrate competition assay used to define the TSR phenotype of a translocation catalyst. The TSR phenotype is highly reliable because the TSR parameter is a *constant*, which renders its value independent of several common variables that, particularly in high-throughput screening, may be poorly controlled or only roughly estimated. A change in the TSR phenotype requires an underlying change in transition state stability (or synonymously an underlying change in catalytic specificity, catalytic power, catalytic efficiency, k_cat_/K_m_, or transition state energy barrier) for one or both of the competing substrates. TSR-scanning mutagenesis is thus expected to identify positions in the protein fold that make contributions to transition state stabilization (the essence of catalytic function). The technical simplicity of TSR analysis should enable broad testing of the hypothesis that in carrier proteins the seat of catalytic power will be delocalized along helix-helix interfaces that *dynamically *enhance structural stability by remodelling in synchrony with transition state formation, thereby promoting translocation catalysis in a manner analogous to recently described networks of coupled promoting motions that allow *dynamic *interactions in the protein fold to enhance transition state stability in enzymatic catalysis [1,11].

## Methods

### Strains and plasmids

*E. coli *strain SK35 is a gabP-negative host strain [8]. *E. coli *SK45 is a gabP-negative strain harbouring the expression plasmid, pSCK380 [8]. *E. coli *SK11 expresses a histidine-tagged Cys-less derivative of GabP [5]. *E. coli* SK105 expresses the Cys-less GabP as a GabP-LacZ hybrid from the plasmid pSCK380Z [24].

### Materials

GABA was from Sigma (St. Louis, MO, U.S.A.); NA was from Research Biochemicals International (Natick, MA, U.S.A.); Miller's Luria Broth medium was from Gibco-BRL (Grand Island, NY, U.S.A.); agar and ampicillin were from Fisher Biotech (Fair Lawn, NJ, U.S.A.); bicinchoninic acid protein determination reagents were from Pierce (Rockford, IL, U.S.A.); cellulose acetate filters (0.45 um; 25 mm) were from either Millipore (Bedford, A, U.S.A.) or MicronSep, (cellulosic; 0.45 um, 25 mm) from OSMONICS Inc. (Minnetonka, MN, U.S.A.); [^3^H] nipecotic acid (40 Ci/mmol) was a custom synthesis from Moravek Biochemicals (Brea, CA, U.S.A.); [^14^C]GABA was from Dupont-New England Nuclear (Boston, MA, U.S.A.); Ultima Gold ™ scintillation cocktail was from Packard BioScience (Meriden, CT, U.S.A.); the anti-Penta-His monoclonal antibody was from QIAGEN (Valencia, CA, U.S.A.); the goat anti-mouse alkaline phosphatase antibody was from Kirkegaard and Perry Laboratories (Gaithersburg, MD); isopropyl-β-D-thiogalactopyranoside (IPTG) was from Anatrace (Maumee, OH); Immobilon-P™ transfer membranes (0.45 um) were from Millipore (Bedford, MA, U.S.A.); the chemiluminescence reagent for alkaline phosphatase detections, Western Lightning, was from Perkin-Elmer Life Sciences, Inc. (Boston, MA, U.S.A.

### *E. coli *culture conditions

*E. coli *strains were recovered by streaking glycerol stocks (-80°C) to single colonies on LB agar supplemented with ampicillin (100 μg/ml). LB broth supplemented with ampicillin (100 μg/ml) was inoculated by picking from a single colony and then shaken overnight (16 h) at 37°C. Overnight cultures were diluted 100-fold into fresh medium, shaken for 2 hours at 37°C prior to adding IPTG (0.2 mM), and shaking for two hours more. Cells were then harvested by centrifugation, washed twice with ice-cold KPi Buffer (100 mM potassium phosphate, pH 7.0), and resuspended to 2 mg protein/ml in the same buffer (20 percent of the original culture volume). Cultures treated in this manner are hereafter referred to as *washed cells*. Washed cells were stored on ice, and then equilibrated to 30°C in a heat block (25 minutes) prior to initiating transport reactions. Cultures treated in this manner are hereafter referred to as *prewarmed *cells.

### Transport conditions

Transport reactions were initiated by mixing 20 μl of a 5-fold concentrated substrate stock solution with 80 μl of prewarmed *E. coli *cell suspension. TSR analysis of the single-Cys GabP variant, N302C, was performed using a substrate stock containing 35 μM [^3^H]NA (2.1 μCi/ml) and 15 μM [^14^C]GABA (0.3 μCi/ml). This solution was found to support equal rates of [^14^C] and [^3^H] label accumulation in the Cys-less GabP control strain [5]. TSR analysis of the GabP variant, INS Ala 320, was performed using a substrate stock containing 20 μM [^3^H]NA (1.2 μCi/ml) and 30 μM [^14^C]GABA (0.6 μCi/ml). This solution was found to support equal rates of [^14^C] and [^3^H] label accumulation by the wild type GabP [6], which contain 5 Cys residues.

A 60 or 120 Hz metronome was used to time the reactions, which were rapidly quenched with 1 ml of ice-cold *Stop Solution *(*KP*_*i *_*Buffer *containing 20 mM HgCl_2_), and then vacuum-filtered (0.45 micron pore). The reaction vessel was then rinsed with 1 ml of *Wash Buffer *(*KP*_*i *_*Buffer *containing 5 mM HgCl_2_) and this was applied to the same filter. Finally, 4 ml of the *Wash Buffer *was applied to the filter. The filter was then dissolved in Ultima Gold™ scintillation cocktail and the [^3^H] and [^14^C] radioactivity (disintegrations per minute, dpm) analyzed with a Packard BioScience Tri-Carb 2900 TR liquid scintillation counter using stored Ultima Gold™ quench curves and automatic quench compensation.

### Standard curves for GabP-independent uptake

The GabP-negative *E. coli *strain, SK45, was grown and prepared for transport experiments as indicated above except that a series of different cell suspensions were prepared spanning a range from 20 to 125 percent of that described above. Dual-label transport experiments carried out with these different suspensions produced a linear standard curve for GabP-independent "background uptake" of [^3^H]NA and [^14^C]GABA as a function of protein content. The protein content of GabP-positive test strains could then be used to obtain the appropriate background subtraction by extrapolation from the standard curve. Test strain protein contents were always similar (within 10 percent) because when cell pellets were resuspended steps were taken to assure approximately equal turbidity levels.

### Statistics

Replicate (n = 3), background-corrected, dual-substrate uptake velocities (moles/time) were inferred from measured disintegration rates for filter-bound [^3^H]NA and [^14^C]GABA. The background-corrected velocity replicates were used to calculate replicate TSR values (Equation 6) from which the mean TSR and standard errors (S.E.M.) shown in the figures were obtained.

### Plasma membrane vesicle preparation and immunoblotting

*E. coli *cells were probe-sonicated to produce plasma membrane vesicles, which were then separated from soluble components and unbroken cells by differential centrifugation as previously described [5]. Plasma membrane proteins were resolved by SDS-PAGE, and transferred to PVDF membranes, which were blocked and then probed with a primary antibody (anti-polyhistidine monoclonal) and secondary antibody (anti-mouse conjugated to alkaline phosphatase) as previously described [5]. Immunoblots were developed with a chemiluminescent alkaline phosphatase substrate (Western Lightning™), and imaged with a cooled CCD camera (Kodak Image Station 440 CF). Chemiluminescent intensities were quantified with Kodak 1D software.
